# Supplementary material for: Vernalisation-induced changes to the Arabidopsis circadian clock require Polycomb Repressive Complex 2 and are FLC-independent
Source: Plant Cell Physiol. Author manuscript; Available in PMC 2026 Oct 16. (PMC7619567; doi:10.1093/pcp/pcag088)
Supplement: Supplementary file 1 [file EMS220400-supplement-Supp_File.zip › Williams et al Supplementary Material v6.pdf]

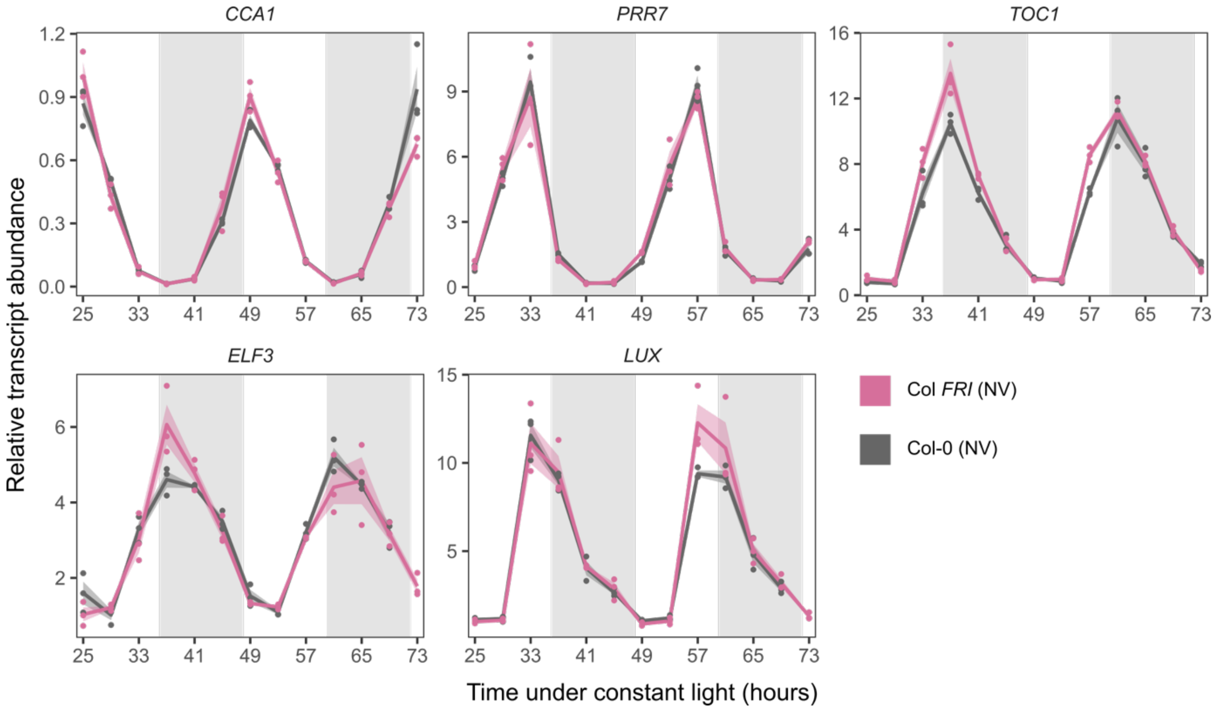

Supplementary Figure 1. Transcript dynamics of circadian oscillator components in non-vernalised Col FRI and Col-0 plants. Relative transcript abundance of circadian oscillator transcripts *CCA1*, *PRR7*, *TOC1*, *ELF3* and *LUX* in non-vernalised Col *FRI* and Col-0 plants. Arabidopsis seedlings were grown under control temperature conditions (12L:12D 19 ˚C) for 26 before being transferred to constant light conditions. Sampling of aerial tissue for RNA started after 25 h of constant light with tissue collected every 4 hours for 48 hours. For each timepoint, aerial tissue from up to five individual plants was pooled to generate each biological replicate. Solid lines indicate mean, with shaded ribbon ± s.e.m (n = 3). Grey shading indicates subjective night. NV = not vernalised.

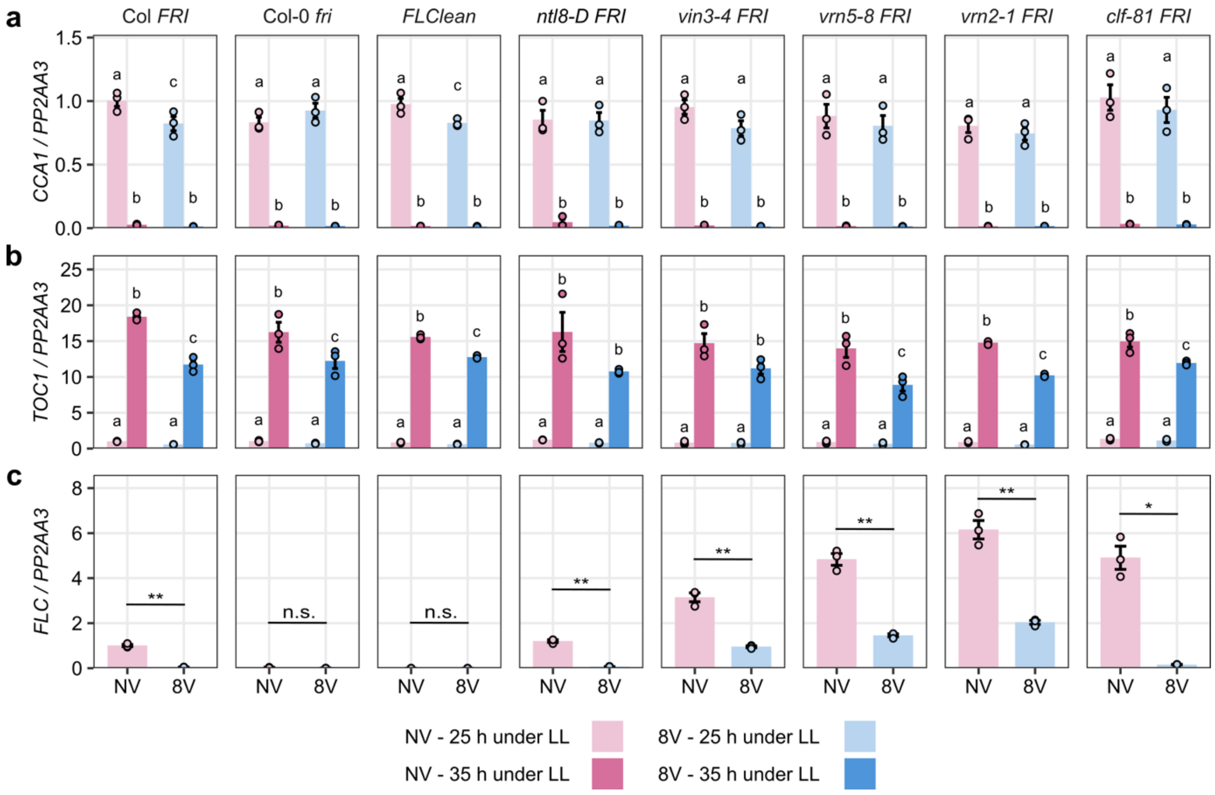

Supplementary Figure 2: Effect of vernalisation pathway mutants upon *CCA1* and *TOC1* transcript levels. **(a, b)** Relative abundance of key circadian oscillator transcripts *CCA1* and *TOC1* in following a period of prolonged cold (8V), compared with non-vernalised (NV) control plants collected following 25 or 35 hours under constant light (LL). Different letters indicate statistically significant differences between transcript levels within each plot ($p < 0.05$; ANOVA with post-hoc Tukey's HSD tests for pairwise comparisons). **(c)** Relative abundance of *FLC* transcripts in NV and 8V plants measured at after 25 h under constant light. Asterisks indicate statistical comparison between vernalised and non-vernalised plants within each genotype (Student's t-test; * $p < 0.05$, ** $p < 0.01$, n.s. $p >= 0.05$). Transcript levels calculated relative to *PP2AA3*, using RT-qPCR. Data are mean ± s.e.m, n = 3.

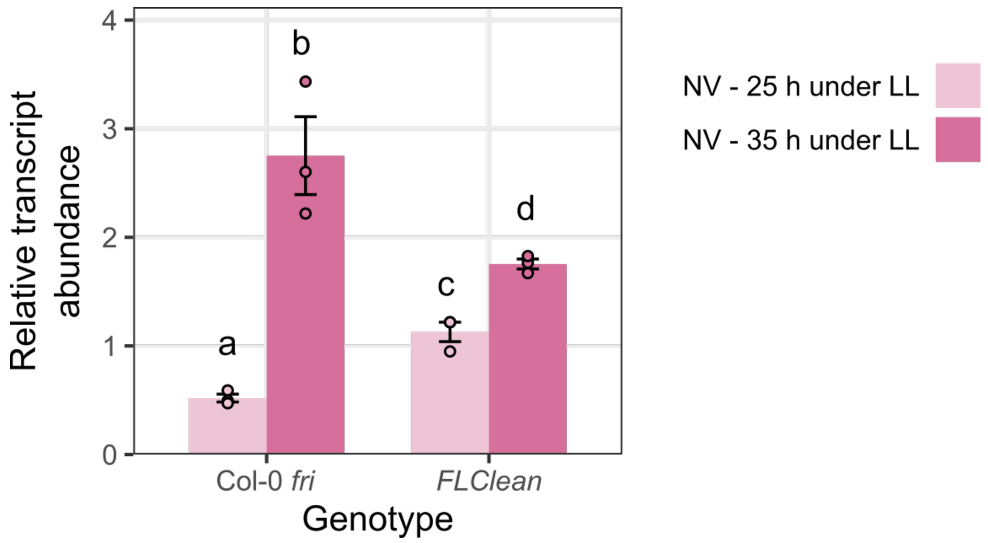

Supplementary Figure 3. Relative transcript abundance of *PRR7* in Col-0 and *FLClean* in non-vernalised plants. Transcript levels calculated relative to *PP2AA3*. Different letters indicate statistically significant differences between transcript levels ($p < 0.05$; ANOVA with post-hoc Tukey's HSD tests for pairwise comparisons). Data are mean ± s.e.m, n = 2-3.

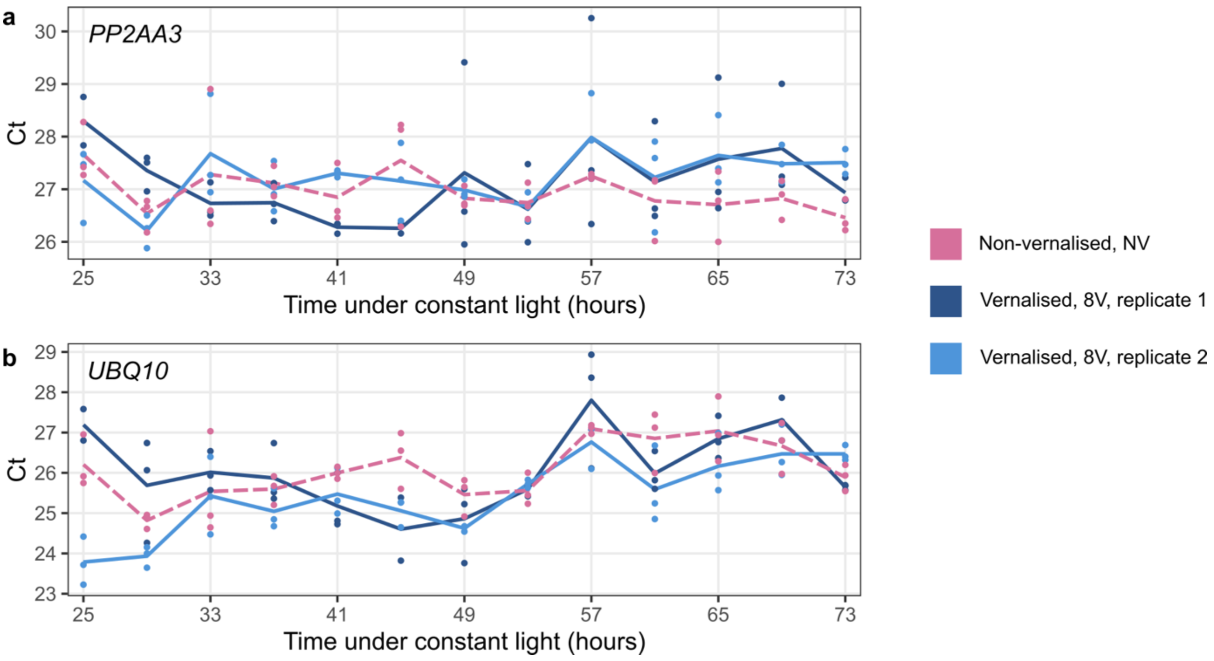

Supplementary Figure 4. Inspection of reference transcripts. Reference transcripts **(a)** *PP2AA3* and **(b)** *UBQ10* were not rhythmic during time courses in our experiments, determined by JTK_CYCLE ($p > 0.5$). Line indicates mean Cp value ($n = 2$-3).

Gene	Chromosome
FRI	4
PRR7	5
CCA1	2
LHY	1
TOC1	5
PRR5	5
LUX	3
ELF3	2
ELF4	2
PRR9	2
CHE	5
HY5	5
HYH	3
COP1	2
GI	1
FLC	5
ZTL	5
PIF4	2
PHYA	1
PHYB	2
CRY1	4
CRY2	1

Supplementary Table 1. Chromosomal locations of key circadian clock-associated genes. *FRI* positioned from nt 268,770 to nt 271,632 on chromosome 4; *CRY1* is positioned from nt 5,723,472 to 5,727,451 on chromosome 4 (data from TAIR 12).

Primer	Purpose	Primer sequence (5' – 3')
ABA1-F	RT-qPCR	AGAGCAACACGGAATTTTCC
ABA1-R	RT-qPCR	TCGATTTCGGAGTTTTCCTG
CBF1-F	RT-qPCR	CCGCCGTCTGTTCAATGGAATCAT
CBF1-R	RT-qPCR	TCCAAAGCGACACGTCACCATCTC
CBF2-F	RT-qPCR	AACTCCGGTAAGTGGGTGTG
CBF2-R	RT-qPCR	CGGCGTATAAATAGCCTCCA
CCA1-F	RT-qPCR	TCTGTGTCTGACGAGGGTCG
CCA1-R	RT-qPCR	ACTTTGCGGCAATACCTCTCTGG
CO-F	RT-qPCR	CTACAACGACAATGGTTCCATTAC
CO-R	RT-qPCR	CAGGGTCAGGTTGTTGC
ELF3-F	RT-qPCR	ACCGAGATGGTGGCAAAACT
ELF3-R	RT-qPCR	ACTGCCATGACCCTCTTGTG
FER3-F	RT-qPCR	TCCAAGAACGATGATGTCCA
FER3-R	RT-qPCR	CCATGTCCTTTGCCAAGTCT
FKF1-F	RT-qPCR	GGATTGTACAACCTTCCCGC
FKF1-R	RT-qPCR	ACCCCTTCCCCACCAAATAG
HY5-F	RT-qPCR	GTCATCAAGCTCTGCTCCACAT
HY5-R	RT-qPCR	CGGCACTCGCCGTATCTC
LUX-F	RT-qPCR	TAACGTGGAGGAGGAAGATCGA
LUX-R	RT-qPCR	TCCATCACCGTTTGATGTCTTT
PIF4-F	RT-qPCR	GCCGATGGAGATGTTGAGAT
PIF4-R	RT-qPCR	CCAACCTAGTGGTCCAAACG
PIF5-F	RT-qPCR	AAAACCCGGTACAGTTGCAG
PIF5-R	RT-qPCR	ATCCAAATCCCAACATGTCC
PP2AA3-F	RT-qPCR	CGTTACTGCCAGCCATTGTA
PP2AA3-R	RT-qPCR	GCAAAGAGCACCAAGCTTCT
PRR7-F	RT-qPCR	CCACGAGCGGTATCTCTATGG
PRR7-R	RT-qPCR	ACTTGGAAACTCAGGGTTAGAATGTAC
PRR9-F	RT-qPCR	AGCATGAAGCTTGCAAGAAC
PRR9-R	RT-qPCR	GCAGCACCTCTCAGCATACA

RVE2-F	RT-qPCR	TCCTTTCGAATGAAATGCTTG
RVE2-R	RT-qPCR	TCTATGGCAGAGCTTGGAGAC
STO-F	RT-qPCR	CCATCCACGTGGCTAATTCT
STO-R	RT-qPCR	AGCCTTCTGTTGGTTGTTGG
TOC1-F	RT-qPCR	TCTTCGCAGAATCCCTGTGAT
TOC1-R	RT-qPCR	GCTGCACCTAGCTTCAAGCA
UBQ10-F	RT-qPCR	GGCCTTGTATAATCCCTGATGAATAAG
UBQ10-R	RT-qPCR	AAAGAGATAACAGGAACGGAAACATAGT

Supplementary Table 2: Primers used in this study.
