# Supplementary material for: Vernalisation-induced changes to the Arabidopsis circadian clock require Polycomb Repressive Complex 2 and are FLC-independent
Source: Plant Cell Physiol. Author manuscript; Available in PMC 2026 Oct 16. (PMC7619567; doi:10.1093/pcp/pcag088)
Supplement: Supplementary file 1 [file EMS220400-supplement-Supp_File.zip › Supplementary Datasets.docx]

**Supplementary Datasets**

**Supplementary Dataset 1.** Quantification of oscillations of circadian clock transcripts before and after vernalisation**.** Estimates of rhythmic parameters of circadian oscillator components in non-vernalised (NV) and vernalised (8V) Col *FRI* plants entrained under 19 ˚C 12L:12D released into free-running conditions.

**Supplementary Dataset 2:** Potential candidate genes that may mediate the vernalisation dependent phase shift of *PRR7* transcripts in Col *FRI*.

**Supplementary Dataset 3:** Source data for this study.
